# Efficacy and safety of ruxolitinib in patients with myelofibrosis: a retrospective and multicenter experience in Turkey

**DOI:** 10.3906/sag-1812-70

**Published:** 2021-06-28

**Authors:** Nur SOYER, Rıdvan ALİ, Mehmet TURGUT, İbrahim C. HAZNEDAROĞLU, Fergün YILMAZ, İsmet AYDOĞDU, Ali PİR, Volkan KARAKUŞ, Gökhan ÖZGÜR, Cem KİS, Funda CERAN, Gül İLHAN, Melda ÖZKAN, Müzeyyen ASLANER, İdris İNCE, İrfan YAVAŞOĞLU, Füsun GEDİZ, Mehmet SÖNMEZ, Birol GÜVENÇ, Gülsüm ÖZET, Emin KAYA, Filiz VURAL, Fahri ŞAHİN, Mahmut TÖBÜ, Raika DURUSOY, Güray SAYDAM

**Affiliations:** 1 Department of Hematology, Faculty of Medicine, Ege University, İzmir Turkey; 2 Department of Hematology, Faculty of Medicine, Uludağ University, Bursa Turkey; 3 Department of Hematology, Faculty of Medicine, Ondokuz Mayıs University, Samsun Turkey; 4 Department of Hematology, Faculty of Medicine, Hacettepe University, Ankara Turkey; 5 Department of Hematology, Atatürk Research and Training Hospital, İzmir Turkey; 6 Department of Hematology, Faculty of Medicine, Celal Bayar University, Manisa Turkey; 7 Department of Hematology, Faculty of Medicine, Karadeniz Technical University, Trabzon Turkey; 8 Department of Hematology, Faculty of Medicine, Muğla Sıtkı Koçman University, Muğla Turkey; 9 Department of Hematology, Gülhane Research and Training Hospital, Ankara Turkey; 10 Department of Hematology, Faculty of Medicine, Çukurova University, Adana Turkey; 11 Department of Hematology, Ankara Numune Research and Training Hospital, Ankara Turkey; 12 Department of Hematology, Faculty of Medicine, Hatay Mustafa Kemal University, Hatay Turkey; 13 Department of Hematology, Faculty of Medicine, İnönü University, Malatya Turkey; 14 Department of Hematology, Dr. Ersin Arslan Research and Training Hospital, Gaziantep Turkey; 15 Department of Hematology, Faculty of Medicine, Adnan Menderes University, Aydın Turkey; 16 Department of Public Health, Faculty of Medicine, Ege University, İzmir Turkey

**Keywords:** Myelofibrosis, treatment, survival, ruxolitinib, adverse events

## Abstract

**Background/aim:**

The aim of this study is to assess the efficacy and safety of ruxolitinib in patients with myelofibrosis.

**Materials and methods:**

From 15 centers, 176 patients (53.4% male, 46.6% female) were retrospectively evaluated.

**Results:**

The median age at ruxolitinib initiation was 62 (28–87) and 100 (56.8%) of all were diagnosed as PMF. Constitutional symptoms were observed in 84.7%. The median initiation dose of ruxolitinib was 30 mg (10–40). Dose change was made in 69 (39.2%) patients. Forty seven (35.6%) and 20 (15.2%) of 132 patients had hematological and nonhematological adverse events, respectively. The mean spleen sizes before and after ruxolitinib treatment were 219.67 ± 46.79 mm versus 199.49 ± 40.95 mm, respectively (p < 0.001). There was no correlation between baseline features and subsequent spleen response. Overall survival at 1-year was 89.5% and the median follow up was 10 (1–55) months. We could not show any relationship between survival and reduction in spleen size (p = 0.73).

**Conclusion:**

We found ruxolitinib to be safe, well tolerated, and effective in real-life clinical practice in Turkey. Ruxolitinib dose titration can provide better responses in terms of not only clinical benefit but also for long term of ruxolitinib treatment.

## 1. Introduction

Myelofibrosis (MF) is a chronic myeloproliferative neoplasm that is characterized by extensive fibrosis of the bone marrow, splenomegaly, and constitutional symptoms such as night sweating, weight loss, and fever [1,2]. MF can present as a primary (PMF) or secondary disease after essential thrombocythemia (ET) or polycythemia vera (PV) [2,3]. Prognosis is assessed by the several prognostic scoring systems such as the International Prognostic Scoring System (IPSS), dynamic-IPSS (DIPSS), and DIPSS-plus [1]. Life expectancy ranges between 2–11 years according to IPSS [1]. 

Janus kinase 2 (JAK2) is a member of intracellular, nonreceptor tyrosine kinases (JAK family) that transduce cytokine mediated signals via the JAK-signal transducer and activator of transcription (STAT) pathway [4,5]. About 65% of MF patients carry a gain of function mutation in JAK2 gene (JAK2 V617F) [1]. Mutations in the calreticulin and The myeloproliferative leukemia virus oncogene (MPL) gene, which also lead to dysregulated JAK/STAT signaling, is identified in patients who do not have the JAK2 mutation [1]. Ruxolitinib is an oral JAK1/JAK2 inhibitor that has demonstrated significant improvement in splenomegaly and constitutional symptoms, and an increase in overall survival (OS) in COMFORT-I and COMFORT-II studies in patients with MF [6–10]. Therefore, ruxolitinib was approved by FDA for the treatment of intermediate- and high-risk MF patients. The JUMP trial which was constructed as phase 3b expanded access trial, covers patients in countries without access to ruxolitinib outside of a clinical study and it also includes patients who classified as intermediate-1 risk. In this study, 62% of patients achieved a > 50% of reduction from baseline of palpable spleen length. The most common adverse events were anemia, thrombocytopenia, primarily grade 1/2 diarrhea, pyrexia, fatigue, asthenia, and infections [11].

In the literature, there are some reports that evaluated the efficacy and safety of ruxolitinib outside of clinical trials in patients with MF. These studies reported reduction in spleen size and improvement of constitutional symptoms [12–14].

We designed this multicenter study to retrospectively evaluate the efficacy and safety of ruxolitinib in Turkish patients with MF in the real-life clinical practice.

## 2. Material and methods

### 2.1. Patients

This study was designed as a retrospective, multicenter study from Turkey, and approved by Ege University Ethical Committee with number of 16–6.1/9. Across all of Turkey, 15 centers were enrolled in the study. We reviewed the medical records of 176 patients with MF from December 2012 to November 2017. The primary objective of the study was the evaluation of the efficacy and safety of ruxolitinib in patients with myelofibrosis in Turkey.

Patients’ ≥ 18 years of age who diagnosed PMF, post-PV or post-ET MF were classified as intermediate- or high risk disease stratified based on the DIPSS or DIPSS plus were included in this study [15,16]. Diagnosis was confirmed according to the 2008 World Health Organization (WHO) criteria and post-PV or -ET MF was diagnosed in accordance with the criteria of the International Working Group of Myelofibrosis Research and Treatment [3,17].

### 2.2. Treatment

In Turkey, ruxolitinib was firstly obtained from the official compassionate use program approved by the Turkish health authorities since October 2011. In June 2016, the commercial use of ruxolitinib was approved and started, and compassionate use program was ended. Patients were only allowed to enter this program if they had a platelet count of 100.000/mm3 or higher. All patients provided written informed consent before the use of this drug. The initial dosage was determined by the physicians according to patient’s clinical condition and platelet count at baseline, ranging from 5 to 20 mg twice daily. The dosage was adjusted every 2–4 weeks based on the platelet counts and the severity of nonhematological toxicities if existed.

A special case report form was used for data collection. Patients’ age of diagnosis, sex, constitutional symptoms, spleen size before and after ruxolitinib treatment, and other clinical parameters were noted. We also collected data of side effects, leukemic evolution, and death.

Response to treatment was reported by the primary treating physicians and analyzed as categorical variables (yes or no for constitutional symptom improvement and spleen size reduction), and as a continuous variable (decrease in spleen size). Spleen length was assessed with abdominal ultrasonography or palpation on physical examination.

### 2.3. Statistical analysis

All the statistical analyses were performed by using the data obtained from the patients’ files. Demographic and disease characteristics of the patients were summarized for all patients using descriptive statistics.

Statistical analyses were performed using SPSS v.16.0 (SPSS Inc., Chicago, IL, USA). The variables were first assessed by Kolmogorov–Smirnov testing in terms of normal distribution. The results were provided as mean ± SD for normally distributed variables and as median (min-max) for nonnormally distributed parameters. Categorical and continuous variables were compared with chi-square and Mann–Whitney U tests, respectively. Logistic regression analysis was performed to correlate spleen response and constitutional symptom response with several baseline features, such as, age > 65 years, sex, diagnostic subgroups, DIPSS score, leukocytosis (> 25.000/µL), hemoglobin < 10 g/dL, platelet < 200.000/µL, blast cells > 1%, JAK2V617F mutation status, and time between diagnosis to ruxolitinib treatment. All p-values were two-tailed and statistical significance was set at the level of p < 0.05.

Overall survival (OS) was defined from the date of ruxolitinib start to the time of death or last follow-up. OS evaluation was performed using the Kaplan–Meier method.

## 3. Results

### 3.1. Baseline characteristics

From 15 centers, 176 patients (94 male; 53.4%, 82 female, 46.6%) were enrolled the study. Patient demographic information and baseline clinical characteristics were shown in Table 1. The median age at diagnosis and ruxolitinib initiation was 59 years (range: 19–83) and 62 years (range: 28–87), respectively. The median interval between diagnosis and the initiation of ruxolitinib was 41.5 months (range: 0–342). One hundred of patients (56.8%) were diagnosed with PMF, 47 (26.7%) and 29 (16.5%) of patients were post-PV MF and post-ET MF, respectively. Hepatomegaly was detected in 89/171 of the patients (52%) and constitutional symptoms were observed in 149/176 (84.7%). Pruritus and minor neurological symptoms were observed in 26.1% and 20.5% of the patients, respectively. Thrombosis (8 arterial, 14 venous, and 2 had both arterial and venous thrombosis) before diagnosis was detected in 13.1%, whereas bleeding was a more rare complication before diagnosis affected 6.3% of the patients. Most common sites of bleeding were epistaxis, gastrointestinal, and cerebral hemorrhage reported in 5, 2, and 2 patients, respectively. Gingival and urinary hemorrhage was observed in 2 patients. Only 2 patients (1.1%) had concomitant cancer history. These were cholangiocellular cancer in one patient and gastric cancer in the other patient. One-hundred and thirteen of all patients (64.2%) were positive for the JAK2 mutation. Eighty patients’ blood analyzed for the MPL mutation. MPL mutation was detected in 6 (7.5%) of 80 patients. Conventional cytogenetic analysis was applied in 31 patients. Twenty three patients had normal karyotypes and 8 had complex karyotypes.

**Table 1:** Patient demographics and baseline clinical characteristics.

Characteristics	n = 176
The median age of ruxolitinib treatment, years (range)	62 (28–87)
Sex, n (%)	
Male	94 (53.4)
Female	82 (46.6)
Myelofibrosis subtypes, n (%)	
PMF	100 (56.8)
Post-PV MF	47 (26.7)
Post-ET MF	29 (16.5)
DIPSS risk category, n (%)	137
Intermediate-1	44 (32.1)
Intermediate-2	80 (58.4)
High	13 (9.5)
DIPSS-plus risk category, n (%)	39
Intermediate-1	4 (10.3)
Intermediate-2	19 (48.7)
High	16 (41)
Hepatomegaly (yes/no/NA) (%)	89/82/5 (50.6/46.6/2.8)
Spleen size before ruxolitinib (mm) (mean ± SD )	219.67 ± 46.79
Constitutional symptoms (yes/no) (%)	149/27 (84.7/15.3)
White blood cell (´ 103/µL) (range)	11 (0.8–68.9)
Platelet (´103/µL) (range)	348 (42–1920)
Hemoglobin (g/dL) (range)	10.7 (6.6–14.9)
Time between diagnosis to ruxolitinib, months, median (range)	41.5 (0–342)
JAK2 mutation (yes/no/NA) (%)	113/55/8 (64.2/31.3/4.5)
Karyotype (yes/no) (%)	31/145 (17.6/82.4)
Peripheral blood blast (%) (range)	0 (0–8)
RBC transfusion history (yes/no), n (%)	108/68 (61.4/38.6)

Antiplatelet, androgen, and steroid treatments were used in 94 (53.4%), 18 (10.2%), and 11 (6.3%) patients, respectively. Seven patients (4%) had history of erythrocyte stimulating agent treatment. Splenectomy and stem cell transplantation was rarely used, in 7 (6%) and 1 patient, respectively.

Cytoreductive treatment was used in 153 (86.9%) patients. The most common drug as the first line treatment was hydroxyurea (131 of 153 patients, 85.6%). Twelve (7.8%) of 153 received anagrelide therapy, 8 (5.2%) interferon and 2 (1.3%) thalidomide. Second line treatment was used in 41 (23.3%) patients. Anagrelide and interferon were the most common second line treatment agents (17 and 15 patients, respectively). Only 8 patients (4.5%) had third line treatment. The most common agent was interferon (n = 5).

### 3.2. Ruxolitinib treatment

The median initial dose of ruxolitinib was 30 mg per day (range:10–40 mg).The initial ruxolitinib doses were 20 mg BID, 15 mg BID, 10 mg BID and 5 mg BID in 67 (38.1%), 36 (20.5%), 46 (26.1%), and 27 (15.3%) patients, respectively. Ruxolitinib dose modifications were necessary in 46% of 150 patients. Ruxolitinib treatment details were given in Table 2. After dose modifications, the median ruxolitinib doses achieved was still 30 mg per day (range: 10–40 mg). The maximum ruxolitinib doses achieved were 20 mg BID, 15 mg BID, 10 mg BID and 5 mg BID in 52 (29.5%), 55 (31.3%), 62 (35.2%), and 7 (4%) patients, respectively.

**Table 2:** Ruxolitinib treatment.

Treatment details	Data
The median initial dose of ruxolitinib (mg)	30 (1–40)
Ruxolitinib dose modification (yes/no) (%) (n = 150)	69/81 (46/54)
The median duration of ruxolitinib (months)	12 (1–52)
Improvement of constitutional symptoms after ruxolitinib (yes/no) (%) (n = 152)	136 / 16 (89.4/10.6)
Improvement of spleen size after ruxolitinib treatment (yes/no) (%) (n = 150)	102/48 (68/32)
Spleen size after ruxolitinib treatment (mm)	199.49 ± 40.95

### 3.3. Efficacy of treatment

Data of improvement in constitutional symptoms and spleen response were available in 152 (86.3%) and 150 (85.2%) patients, respectively. Improvement in constitutional symptoms was seen in 136/152 (89.4%) patients. A reduction in splenomegaly was seen in 102/150 (68%) patients.

The mean spleen sizes before and after ruxolitinib treatment were 219.67 ± 46.79 mm versus 199.49 ± 40.95, respectively (p < 0.001). A ≥ 50% reduction from baseline in palpable spleen length was seen in 17/39 (43.5%) patients at any time during the study. Mean percentage change from baseline to week 12 in ultrasonographic spleen length showed in Figure 1. Mean percentage change from baseline in palpable spleen length showed in Figure 2. Among baseline features that were tested for correlation with subsequent spleen response and constitutional symptom response, none were significantly associated with these. The data showed in Table 3. There was no correlation between maximum ruxolitinib doses achieved and reduction in spleen size. 

**Table 3:** Logistic regression analysis between baseline factors, spleen, and constitutional symptom responses.

	OR	95% CI	p-value
Spleen response			
Age > 65 years	0.88	0.30–2.58	0.82
Sex (male)	1.14	0.46–2.81	0.76
Post-PV MF	1.63	0.84–3.15	0.14
DIPSS intermediate-1	0.70	0.22–2.22	0.55
Leukocytosis > 25.000/µL	1.21	0.30–4.89	0.78
Hemoglobin < 10 g/dL	2.44	0.84–7.07	0.09
Platelet < 200.000/µL	1.41	0.49–4.02	0.51
Blast cells > 1%	1.62	0.54–4.83	0.38
JAK2V617F mutation positive	1.02	0.36–2.91	0.95
Time between diagnosis to ruxolitinib treatment > 2 years	1.00	0.99–1.01	0.80
Constitutional symptoms response			
Age > 65 years	4.35	0.44–42.51	0.20
Sex male	0.88	0.19–3.95	0.86
Post-PV MF	0.11	0.11–1.91	0.06
DIPSS intermediate-1	2.16	1.30–35.7	0.32
Leukocytosis (> 25.000/µL)	0.52	0.06–4.21	0.54
Hemoglobin < 10 g/dL	1.39	0.28–6.75	0.68
Platelet < 200.000/µL	1.52	0.33–6.86	0.58
Blast cells > 1%	2.19	0.36–13.04	0.38
JAK2V617F mutation positive	0.16	0.01–1.68	0.08
Time between diagnosis to ruxolitinib treatment > 2 years	0.98	0.96–2.99	0.39

### 3.4. Safety and outcome

Adverse events data were available in 132 (75%) of all patients. Forty seven (35.6%) of 132 patients had hematological and 20 (15.2%) had nonhematological adverse events. Adverse events are illustrated in Table 4. Patients who had anemia treated with red blood cell transfusions. For management of thrombocytopenia, ruxolitinib dose reduction was performed. Nonhematological adverse events (AST-ALT elevation, abdominal pain, rash, nausea, gingival bleeding, and electrolyte imbalance) were treated with supportive care. Infections were treated with antimicrobial therapy.

**Table 4:** Adverse events.

	All grades, n = 132 (%)
Hematological adverse events	
Anemia	32 (24.2)
Thrombocytopenia	25 (18.9)
Neutropenia	1 (0.75)
Nonhematologic adverse events	
AST-ALT elevation	4 (3)
Fatigue	3 (2.3)
Urinary tract infection	3 (2.3)
Abdominal pain	2 (1.5)
Pneumonia	2 (1.5)
Zona zoster	2 (1.5)
Dizziness	1 (0.75)
Gingival bleeding	1 (0.75)
Rash	1 (0.75)
Palpitation	1 (0.75)
Electrolyte imbalance	1 (0.75)
Nausea	1 (0.75)

Overall, 26 patients (14.8%) died because of leukemic transformation (n = 3), cardiac diseases (n = 4), pneumonia/sepsis (n = 8), acute respiratory distress syndrome (n = 1), cholangiocellular cancer (n = 1), bleeding (n = 3), and disease progression without leukemic transformation (n = 6). Death occurred after a median ruxolitinib exposure of 9.4 months (1–45.1 months); in no case the death was directly attributed to therapy. Estimated OS at 1-year was 89.5% and the median follow up was 10 (1–55) months, as shown in Figure 3. Estimated OS at 3-year from ruxolitinib start was 72.3% in patients achieving a spleen response and 68.3% in patients without a spleen response. The mean OS was 45.05 ± 2.8 months in patients who had reduction in spleen size, whereas the mean OS was 38.24 ± 4.1 months in patients who had no reduction in spleen size. Statistical significance was not observed between OS and spleen response (p = 0.73), as shown in Figure 4.

## 4. Discussion

The aim of this retrospective and multicenter study was to evaluate the efficacy and safety of ruxolitinib in Turkish patients with MF in the real-life clinical practice. We evaluated 176 patients from 15 centers across all of Turkey.

In our study, the median age at ruxolitinib treated patients (62 years) was slightly lower than literature (61.8–74 years) [11,13,14,18–24]. Sex and subtype of myelofibrosis were similar to literature [11,13,18,20,22–24]. Intermediate-2 risk patients (58.4%, n = 137) were the most common prognostic group according to DIPSS in this cohort, this finding was compatible with the literature (30%–62.4%) [13,14]. In COMFORT-I and II trials, patients were intermediate-II and high risk group according to IPSS [6,7]. In our study, 32.1% of 137 patients who received ruxolitinib were intermediate-1 risk according to DIPSS and it was higher than literature [13,14]. This higher rate may be associated with limited effective treatment options for MF in the real life practice. In the literature, rates of IPSS low and intermediate-I risk group patients in real-life studies were between 11.8% and 31% [11,18,22]. One should not forget that most of our patients are from the compassionate use program, which means that they were allowed in special conditions.

Most patients in this study experienced reduction in spleen size (68%) and improvement in constitutional symptoms (89.4%). These results were comparable with previous study reported by Ellis et al. [14]. The rate of patients achieving 50% or more reduction in palpable spleen size in this study was similar to literature [11,14,18–20,22]. In this study, both ultrasonographic and palpable spleen size were evaluated. In the real-life setting, physical examination of a patient is performed by various physicians in outpatient clinics. Ultrasonography is a common, simple, and cheap imaging technique. Although we did not evaluate spleen volume with ultrasonography in all patients, spleen size reduction after ruxolitinib treatment was significant in both physical and ultrasonographic examination.

The median initial dose of ruxolitinib was 30 mg per day in our analysis and this dose was compatible with literature [14,18] but dose modification rate was lower than reported in other studies (46% vs. 54%–88.9%) [11,14,18,22,23]. Lower dose modification rate in our study might be associated with lower initial dose rate than other studies. In our study, 38.1% of patients started at the dose of 20 mg BID, whereas 54.2%–63.6% of patients started at the dose 20 mg BID in other studies [11,18,20,23]. After dose modifications, the median dose of ruxolitinib was still 30 mg per day in this study. There was no correlation between maximum ruxolitinib doses achieved and spleen response.

The most common hematological adverse events were anemia (24.2%) and thrombocytopenia (18.9%) in our study as expected. Anemia was relatively lower than other studies (39.7%–62.7%) [11,18,22]. Thrombocytopenia was slightly lower than others (25.5%–40.5%) [11,18,22]. The most common nonhematological adverse events were abnormal hepatic (3%) function tests and infections (5.3%) in this study. These rates were much lower compared to other studies [11,18,22]. The lower rates of toxicities in our study might be related to lower initial dose of ruxolitinib and close monitorization of patients in terms of toxicities. Since it is retrospective study, adverse event data might have been reported less frequently.

Baseline factors were not associated with spleen response and constitutional symptom response in our study. In a retrospective study, high/intermediate-2 IPSS risk, a large (≥ 10 cm below LCM) splenomegaly, transfusion dependency, platelet count < 200 ´ 109/L, and a time-interval between MF diagnosis and RUX start > 2 years were significantly associated with lower spleen response [20]. The same study was also evaluated pre-treatment factors negatively correlating with symptom response. In multivariate analysis, a baseline total symptom score (TTS) > 20 had a significantly lower probability of achieving a symptoms response at 6 months. In our study, because numbers of intermediate-1 DIPSS risk patients were higher than other studies, more patients who received ruxolitinib had less advanced disease. The lack of correlation between the spleen response and some baseline factors can be explained with higher rate of less advanced disease and relatively small patient population. 

The estimated OS at 1 year was similar to JUMP trial (89.5% vs. 94%) [11]. The estimated OS at 3 years from ruxolitinib start was 72.3% in patients achieving a spleen response and 68.3% in patients without a spleen response. Although these rates were comparable with literature (77.9% in patients achieving a spleen response and 68.4% in patients without a spleen response, p = 0.034), statistical significance was not shown in our study (p = 0.736) [20]. Lower initial dose of ruxolitinib in our study might be associated with slightly lower spleen response. 

There are some limitations of this study. Firstly, this is a retrospective study so, some of the data was not found because of inadequate records. Secondly, a relatively small patient population was included in our study compared to the literature. Thirdly, spleen and constitutional symptoms responses were evaluated with physicians’ reports. Ultrasonography was also used for imaging the spleen response in many centers. But this technique was not routinely used for evaluating spleen response in studies. 

In conclusion, ruxolitinib is a safe and effective therapy in Turkish patients with MF. Indeed lower initial ruxolitinib doses were associated with lower dose modification rate; spleen response might be affected by lower ruxolitinib doses. We can conclude that ruxolitinib dose titration based on the current guidelines can provide better responses in terms of not only clinical benefit but also for long term of ruxolitinib treatment.

## Informed consent

This study was approved by Ege University Ethical Committee with a number of 16-6.1/9.
